# Large next-generation sequencing gene panels in genetic heart disease: yield of pathogenic variants and variants of unknown significance

**DOI:** 10.1007/s12471-019-1250-5

**Published:** 2019-03-07

**Authors:** F. H. M. van Lint, O. R. F. Mook, M. Alders, H. Bikker, R. H. Lekanne dit Deprez, I. Christiaans

**Affiliations:** 1 Department of Clinical Genetics, Amsterdam University Medical Center, University of Amsterdam, Amsterdam, The Netherlands; 2 Department of Clinical Genetics, University Medical Center Groningen, University of Groningen, Groningen, The Netherlands

**Keywords:** Next-generation sequencing, Variants of unknown significance, Cardiogenetic, Gene panel, Detection rate

## Abstract

**Background:**

Genetic heterogeneity is common in inherited cardiac diseases. Next-generation sequencing gene panels are therefore suitable for genetic diagnosis. We describe the results of implementation of cardiomyopathy and arrhythmia gene panels in clinical care.

**Methods:**

We present detection rates for variants with unknown (class 3), likely (class 4), and certain (class 5) pathogenicity in cardiogenetic gene panels since their introduction into diagnostics.

**Results:**

In 936 patients tested on the arrhythmia panel, likely pathogenic and pathogenic variants were detected in 8.8% (4.6% class 5; 4.2% class 4), and one or multiple class 3 variants in 34.8%. In 1970 patients tested on the cardiomyopathy panel, likely pathogenic and pathogenic variants were detected in 19.8% (12.0% class 5; 7.9% class 4), and one or multiple class 3 variants in 40.8%. Detection rates of all different classes of variants increased with the increasing number of genes on the cardiomyopathy gene panel. Multiple variants were detected in 11.7% and 28.5% of patients on the arrhythmia and cardiomyopathy panels respectively. In more recent larger versions of the cardiomyopathy gene panel the detection rate of likely pathogenic and pathogenic variants only slightly increased, but was associated with a large increase of class 3 variants.

**Conclusion:**

Overall detection rates (class 3, 4, and 5 variants) in a diagnostic setting are 44% and 61% for the arrhythmia and cardiomyopathy gene panel respectively, with only a small minority of likely pathogenic and pathogenic variants (8.8% and 19.8% respectively). Larger gene panels can increase the detection rate of likely pathogenic and pathogenic variants, but mainly increase the frequency of variants of unknown pathogenicity.

**Electronic supplementary material:**

The online version of this article (10.1007/s12471-019-1250-5) contains supplementary material, which is available to authorized users.

## What’s new?

- Overall detection rate of likely pathogenic and pathogenic variants and variants of unknown significance using large diagnostic gene panels is 61% and 44% for cardiomyopathies and primary arrhythmia syndromes respectively.
- The majority of detected variants in cardiogenetic gene panels is still classified as variant of unknown significance.
- Larger gene panels can increase the detection rate of pathogenic variants, but mainly increase the frequency of variants with unknown pathogenicity and of multiple variants.

## Introduction

Next-generation sequencing (NGS) techniques with sequencing and analysis of multiple genes in a single experiment are being used more frequently in a diagnostic setting in monogenic diseases. Examples are whole exome sequencing (WES) and disease-specific gene panels which can be complemented by Sanger sequencing to cover the entire coding region of the specified genes. Gene panels are particularly well suited in genetic heart diseases, because of their phenotypic and genetic heterogeneity; a single disease associated with different genes, and a single gene associated with different diseases [1].

Testing more genes using a gene panel not only increases the detection rate of disease-causing variants, but also of so-called variants of uncertain/unknown significance (VUS). VUS are variants whose implication with disease cannot be concluded based on current data. With time and further knowledge, VUS can then be classified as either benign without clinical significance or disease-causing.

This article shows the yield of pathogenic variants and VUS of cardiogenetic gene panels for primary arrhythmia syndromes and cardiomyopathies used in a diagnostic setting. A separate article describes the challenges for counselling and clinical decision making that can arise from unclear gene panel results.

## Methods

### Study population and clinical evaluation

Data were collected from molecular diagnostics of all probands (first patient in their family) in whom a gene panel was performed at the DNA laboratory of Amsterdam UMC, University of Amsterdam. Patients were included from the introduction of gene panels in diagnostic settings (arrhythmia panel: November 2013; cardiomyopathy panel: February 2012) until January 1, 2018. Patients mainly came from the departments of Clinical Genetics of Dutch University hospitals.

In 2,829 probands, genetic testing was performed using the gene panels: 936 probands on the arrhythmia gene panel, 1970 on the cardiomyopathy gene panel and 77 on both panels. The results of a few patients have been published before [2]. Almost all panel requests came from clinical geneticists specialised in cardiogenetics. We collected data on (suspected) clinical diagnosis from the DNA request form: hypertrophic cardiomyopathy (HCM), dilated cardiomyopathy (DCM), arrhythmogenic (right ventricular) cardiomyopathy (ARVC), noncompaction cardiomyopathy (NCCM), unspecified cardiomyopathies (UCM), long QT syndrome (LQTS), Brugada syndrome (BRS), cathecholaminergic polymorphic ventricular tachycardia (CPVT), unspecified arrhythmia (UA) and other indications (ventricular fibrillation, ventricular tachycardia, sudden cardiac death, conduction disorders). Not all patients fulfil diagnostic criteria for the disease as suspicion can be enough reason to be tested. It is beyond the scope of this study to include detailed clinical data of evaluated patients. All patients gave informed consent for the anonymous use of their genetic data.

### Next-generation sequencing (NGS) gene panels

Detailed information on NGS, the platforms used and their analytical performance has been published before and is described in the online Supplementary File 1 [2, 3]. Tab. 1 shows the genes in the different versions of the arrhythmia and cardiomyopathy panels.

**Table 1:** Genes included in the gene panels

Gene panel	Genes	Patients tested (*n*)
Arrhythmia panel version 1 (43 genes)	*ABCC9, AKAP9, ANK2, CACNA1C, CACNA1D, CACNA2D1, CACNB2, CALM1, CALM2, CALM3, CASQ2, CAV3, DPP6 (only position c.-340), GJA5, GPD1L, HCN4, KCNA5, KCND3, KCNE1, KCNE1L, KCNE2, KCNE3, KCNH2, KCNJ2, KCNJ5, KCNJ8, KCNQ1, LAMP2, LMNA, NPPA, PKP2, PLN, PRKAG2, RANGRF, RYR2, SCN1B, SCN3B, SCN4B, SCN5A, SNTA1, TNNT2, TRDN, TRPM4*	170
Arrhythmia panel version 2 (47 genes)	Version 1 *+* *ASPH, JPH2, SCN2B, SLMAP*	90
Arrhythmia panel version 3 (48 genes)	Version 2 *+* *SCN10A*	308
Arrhythmia panel version 4 (49 genes)	Version 3 *+* *NKX2-5*	199 (150 incl. CNV)
Arrhythmia panel version 5 (50 genes)	Version 4 *+* *PPA2*	172
Cardiomyopathy panel version 1 (23 genes)	*ACTC1, CSRP3, DES, EMD, GLA, LAMP2, LDB3, LMNA, MYBPC3, MYH7, MYL2, MYL3, PLN, PRKAG2, SCN5A, SGCD* ^*a*^ *, TAZ, TCAP, TNNC1, TNNI3, TNNT2, TPM1, VCL*	325
Cardiomyopathy panel version 2 (41 genes)	Version 1 *+* *ACTN2, ANKRD1, BAG3, CALR3, CAV3, CRYAB, DSC2, DSG2, DSP, FHL1, JPH2, JUP, MYH6, MYOZ2, MYPN, PKP2, RBM20, TMEM43, TTR*	261
Cardiomyopathy panel version 3 (46 genes)	Version 2 *+* *CTNNA3, LAMA4, MIB1, NEXN, PRDM16*	188
Cardiomyopathy panel version 4 (47 genes)	Version 3 *+* *TTN*	347
Cardiomyopathy panel version 5 (50 genes)	Version 4 *+* *ALPK3, FHL2, HCN4*	603 (206 incl. CNV)
Cardiomyopathy panel version 6 (53 genes)	Version 5 *+* *CDH2, FLNC, PPA2*	246

CNV (copy number variant) analysis was added to the gene panels in January 2017. Thus, not all patients analysed on the arrhythmia panel version 4 and cardiomyopathy panel version 5 have had CNV analysis

^a^The *SGCD* gene was removed from the cardiomyopathy panels version 2 and 3 because of inadequate evidence that the gene is associated with cardiomyopathies

Variants are reported using Human Genome Variation Society nomenclature guidelines (http://www.hgvs.org/mutnomen) and classified into one of five categories (class 1: certainly not pathogenic, class 2: unlikely pathogenic, class 3: unknown pathogenicity, class 4: likely pathogenic; class 5: (certainly) pathogenic) using the classification criteria as indicated in the online Supplementary File 1. Identified class 1 and 2 variants are not reported. Classification was carried out at the moment the sequence data were analysed using the data available at that time. Reinterpretation and reclassification was only done when the variant was detected in another patient or more information became available from the family or other cases in other clinical genetics centres in the Netherlands.

### Statistical analysis

Variables are reported as frequency (%). Categorical and dichotomous variables are compared between groups using the chi-squared test. A two-sided *p*-value of <0.05 was considered significant. SPSS (version 23.0. Armonk, NY: IBM Corp.) was used for all statistical analyses.

## Results

### Arrhythmia gene panel

In 412 of the 936 (44.0%) patients analysed for the arrhythmia gene panel one or more variants were detected (detailed information in Tab. 2 and online Supplementary File 2). Forty-three patients (4.6%) had a pathogenic (class 5) variant, 39 (4.2%) a likely pathogenic (class 4) variant, and 326 (34.8%) had ≥1 class 3 variants. Four patients carried a genetic risk factor (c.253G > A; p.(Asp85Asn)) in *KCNE1*. This specific *KCNE1* variant has an allele frequency of ~1%, but is known to be associated with LQTS [4]. The 82 pathogenic variants (including likely pathogenic) were detected in 16 different genes. Multiple variants were detected in 11.8% of patients, with a maximum of four variants in a single patient.

**Table 2:** Detected variants since 2015 per diagnosis category

Diagnosis	Patients	Variants	Highest pathogenicity class			Multiple	Additional information
		Total	Class 5	Class 4	Class 3		
In the arrhythmia gene panel in 572 patients with the most common indications							
Brugada syndrome	42	24 (57.1%)	4 (9.5%)	0 (0%)	23 (54.8%)	13	
LQTS	65	35 (53.8%)	7 (10.8%)	5 (7.7%)	23 (35.4%)	11	
CPVT	38	23 (60.5%)	0 (0%)	5 (13.2%)	18 (47.4%)	8	
SCA/SCD	6	2 (33.3%)	0 (0%)	0 (0%)	2 (33.3%)	0	
UA	421	190 (45.1%)	17 (4.0%)	16 (3.8%)	155 (36.8%)	48	Two with risk factor (c.253G > A; p.(Asp85Asn) in *KCNE1)*
In the cardiomyopathy gene panel in 1,281 patients with the most common indications							
HCM	453	319 (70.4%)	70 (15.5%)	27 (6.0%)	222 (49.0%)	184	
DCM	396	271 (68.4%)	28 (7.1%)	37 (9.3%)	206 (52.0%)	138	
NCCM	67	44 (65.7%)	7 (10.4%)	5 (7.5%)	32 (47.8%)	21	
ARVC	113	75 (66.3%)	19 (16.8%)	6 (5.3%)	50 (44.2%)	36	
UCM	252	166 (65.9%)	22 (8.7%)	18 (7.1%)	126 (50.0%)	86	

*LQTS* long QT syndrome, *CPVT* cathecholaminergic polymorphic ventricular tachycardia, *SCA/SCD* sudden cardiac arrest/sudden cardiac death, *UA* unspecified arrhythmia, *HCM* hypertrophic cardiomyopathy, *DCM* dilated cardiomyopathy, *NCCM* noncompaction cardiomyopathy, *ARVC* arrhythmogenic right ventricular cardiomyopathy, *UCM* unspecified cardiomyopathies

### Cardiomyopathy gene panel

In 1,194 of the 1970 (60.8%) patients analysed for the cardiomyopathy gene panel, one or more variants were detected (detailed information in Tab. 2 and online Supplementary File 2). 236 patients (12.0%) had a pathogenic variant, 155 (7.9%) ≥1 class 4 variants and 803 (40.8%) ≥1 class 3 variants. The 391 pathogenic variants (including likely pathogenic) were detected in 34 different genes. Multiple variants were detected in 28.5% of patients with a maximum of seven variants in a single patient.

### Yield and gene panel size

Yield of all different classes of variants, but mainly class 3, increased when more genes were analysed on the panel. There was a significant increase in yield of class 3 variants before and after 2015 (including the* TTN* gene since 2015 in the cardiomyopathy panel) for the arrhythmia panel (χ2(2) = 25,133; *p* < 0.01) and cardiomyopathy panel (χ2(2) = 633,987; *p* < 0.01), but not of class 4 and 5 variants (Tab. 3).

**Table 3:** Yield of the arrhythmia and cardiomyopathy gene panel before and after 2015

Pathogenicity class	Number of variants < 2015		Number of variants ≥ 2015	
*Arrhythmia gene panel*				
Class 5	10	(3.9%)	22	(3.2%)
Class 4	11	(4.2%)	28	(4.1%)
Class 3	74	(28.2%)	252	(37.2%)
Risk factor	2	(0.8%)	2	(0.3%)
No variants reported	164	(63.3%)	375	(55.4%)
*Cardiomyopathy gene panel*				
Class 5	86	(11.1%)	150	(12.5%)
Class 4	58	(7.5%)	97	(8.1%)
Class 3	179	(23.2%)	624	(52.1%)
No variants reported	450	(58.2%)	326	(27.2%)

To see if larger gene panels perform better than small disease-specific panels for 508 patients with (suspected) HCM we compared the yield of class 4 and 5 variants of our cardiomyopathy gene panel with that of a fictive gene panel of seven genes with a clear association with HCM (*MYBPC3, MYH7, TNNT2, TNNI3, TPM1, MYL2, MYL3*). In 172 HCM patients a class 4/5 variant was detected. Using this fictive gene panel we would have missed 9 of 122 class 5 variants and 19 of 50 class 4 variants. The fictive gene panel would yield 28.3% class 4/5 variants instead of 33.9% (*p* = 0.0672).

### Dutch founder variants

In the Netherlands, several variants have been detected more frequently. For many of these variants haplotype analysis and genealogy suggest the variant originates from an ancient founder. In 100 patients a previously described founder variant was detected and in 81 another recurrent variant (online Supplementary File 3). Founder variants and recurrent variants made up 34.9% of class 5 variants in the arrhythmia panel and 70.3% of class 5 variants in the cardiomyopathy panel.

### Variant reclassification

We looked at reclassification of variants that occurred between January 1, 2015 and January 1, 2018, based on reinterpretation. Most variants were not reclassified at reinterpretation. For the arrhythmia panel seven variants were reclassified and for the cardiomyopathy panel 13 (upgrade of pathogenicity class in 9, downgrade in 11). Reasons for reclassification were new information on frequency of the variant in control databases (*n* = 6), results of RNA studies (*n* = 5), co-segregation in the family (*n* = 5) and additional patients/families with the same variant (*n* = 4) (online Supplementary File 2).

## Discussion

Overall detection rate (class 3, 4, and 5 variants) is 44% and 61% for the arrhythmia and cardiomyopathy panels respectively, with only a minority of likely pathogenic and pathogenic variants (8.8% and 19.8% respectively). These yields of likely pathogenic and pathogenic variants are lower than published in pre-NGS literature [5–9] and NGS literature (data ≥ 2015: HCM: 21.4% class 4/5 variants in our cohort vs 32% in literature [10]; DCM: 16.3% class 4/5 variants in our cohort vs 37% in literature [11]; Brugada syndrome: 9.5% class 4/5 variants in our cohort vs ~20% in literature [7, 12]). Even the substantial contribution of Dutch founder variants does not raise detection rates. There are several explanations for this. First, indications for diagnostic DNA testing broadened in the past years. It is no longer only patients meeting diagnostic criteria for disease, such as the well-phenotyped research cohorts from literature, who are being tested. A study of our centre also showed decreasing yields in time explained by patients with less severe phenotypes being tested in more recent years [13], and studies have been published confirming that yield is higher in more severe patients [14]. Increased awareness of genetic heart disease also gives rise to more referrals of patients with a merely suspected personal or family history of disease. An unclear phenotype is a likely explanation for the lower yield of the arrhythmia panel, as, for example, sudden cardiac arrest or sudden cardiac death could also have non-monogenic and/or non-cardiac causes (Tab. 2). Patients with a clear diagnosis of LQTS or CPVT, for which a small set of genes can be tested with a high pathogenic yield, are underrepresented in the arrhythmia panel cohort. Second, standards for calling a variant pathogenic have become more stringent. The difference between likely pathogenic and pathogenic was almost absent in early years. Any rare evolutionary conserved variant not found in ~100 healthy controls was considered pathogenic. Third, inherited cardiac diseases are relatively common and show incomplete and age-dependent penetrance, meaning that also pathogenic variants or modifiers can be detected in control databases and can have a relatively high frequency in the general population. Presence of a variant in control databases therefor does not exclude pathogenicity. Whereas for highly penetrant, rare diseases low-frequency variants are labelled likely benign or benign (class 1 or 2 variant), for cardiac diseases they will be classified as unknown pathogenicity (class 3 variant). Fourth, knowledge on many of the genes on the panels and variant classification in these genes is sparse, because clear disease associations have been made in a few patients. Variants are therefore easily classified as a type 3 variant.

The increasing number of genes on different versions of our cardiomyopathy panel did result in a higher detection rate of likely pathogenic and pathogenic variants, but mainly of class 3 variants. This has been shown previously in literature [10, 11, 15]. A lower number of tested genes makes interpretation of test results easier, with less VUS, but also increases the risk of missing pathogenic variants. The addition of the *TTN* gene to the cardiomyopathy panel in 2015 only resulted in an increase of class 4 and 5 variants in the DCM patients, but not in other cardiomyopathy patients. In all patients, however, the addition of *TTN* resulted in an increase of class 3 variants. This can, however, also be the result of less severe patients being tested in more recent years. Developments in diagnostic DNA testing and increasing knowledge on tolerated DNA variants ask for continuous re-evaluation of which test (single genes or large panel) or which filter used for a genome-wide test is best suited for which patient. Initiatives like ClinGen (www.clinicalgenome.org), which evaluates clinical validity of supposed disease genes, can be of help with this, and possibly patients can make an informed choice in the extent of the DNA test and the given results. Although reclassification was uncommon on reinterpretation in our cohort, updates of classification status of class 3–5 variants should be made regularly, including reporting important reclassifications back to the physician who requested the test. Initiatives that allow sharing of variant data could help in solving the meaning of observed VUS.

Multiple variants were detected in 11.8% and 28.5% of patients on the arrhythmia and cardiomyopathy panels respectively. Some variants will eventually be classified as benign variants, but others will be associated with disease. Although inherited cardiac diseases are considered to follow an autosomal dominant inheritance pattern, the presence of multiple variants in our study, and previous descriptions of multiple rare variants, digenic inheritance, homozygous and compound heterozygous variants, all suggest more complex inheritance patterns and modifier effects [16–19]. This might also explain the variable disease expression of inherited cardiac diseases.

The high frequency of VUS in cardiogenetic gene panels necessitates pre-test counselling on VUS of tested patients. However, incidental findings can still give rise to challenges in counselling/clinical decision making. In a separate article we describe these challenges in more detail. We recommend pre-test and post-test counselling to be performed by a physician/counsellor with sufficient knowledge on VUS and variant classification. Challenging cases should be discussed in a multidisciplinary cardiogenetics team.

### Conclusions

Overall detection rates for cardiogenetic NGS gene panels in a diagnostic setting are 44% and 61% for the arrhythmia and cardiomyopathy gene panels respectively, with only a small minority of likely pathogenic and pathogenic variants (8.8% and 19.8% respectively). Larger gene panels can increase detection rates of likely pathogenic and pathogenic variants, but mainly increase the frequency of VUS. Test results, especially VUS and incidental findings, can be challenging for genetic counselling and ask for proper pre-test and post-test counselling and evaluation by a multidisciplinary cardiogenetics team.

## Caption Electronic Supplementary Material

**Supplementary File 1** Supplementary methods

**Supplementary File 2** Detected variants in the panels

**Supplementary File 3** Detected known Dutch founder variants and other recurrent possible founder variants (all class 5)
