# Supplementary material for: Large next-generation sequencing gene panels in genetic heart disease: yield of pathogenic variants and variants of unknown significance
Source: Neth Heart J. 2019 Mar 7;27(6):304–9. doi: 10.1007/s12471-019-1250-5 (PMC6533346; doi:10.1007/s12471-019-1250-5)
Supplement: Supplementary file 1 — Supplementary methods [file 12471_2019_1250_MOESM1_ESM.doc]

**Online Supplementary File 1** Supplementary methods

**Next-generation sequencing (NGS)**

All next-generation sequencing methods used were performed as a type A test (>99% reliable reference or variant calls of the coding region and flanking intronic sequences). All coding exons of the genes, including the 20 flanking intron nucleotides have been analysed. Copy number variation of the analysed regions was included in the analyses in January 2017. Copy number variation (CNV) detection was introduced in arrhythmia panel version 4 and cardiomyopathy panel version 5. CNV detection is calculated by means of Depth of Coverage (DoC) per exon after going through the BWA-MEM-GATK pipeline. DoC per exon per sample is normalised to average coverage per sample and compared to average normalised coverage per exon of previously analysed samples. Differences in normalised coverage are used to calculate Z-scores.

Target enrichment was initially done with the use of custom designed microarrays (Nimblegen) and later with custom designed in solution captures (SeqCap EZ Choice, Nimblegen) using Nimblegen Rebal algorithm. Samples on the cardiomyopathy panel version 1 (23 genes) were all sequenced with the GS-FLX Titanium (Roche). Samples on the arrhythmia panel version 1 and cardiomyopathy panel version 2 were sequenced on the SOLiD 5500 (Life Technologies) and the MiSeq (Illumina). Samples on subsequent versions of the arrhythmia and cardiomyopathy panels were all sequenced on the MiSeq. SOLiD 5500 paired-end sequencing reads (75 x 35 bp) were mapped in colour space to the GRCh37/hg19 reference genome using LifeScope software version 2.5.1 (Life Technologies). Variants were identified using the HaplotypeCaller from GATK version 2.4.3 (Genome Analysis Toolkit, Broad Institute) along with Picard tools version 1.89. MiSeq paired-end sequencing reads (2 x 150 bp) were mapped to GRCh37/hg19 reference genome using BWA-MEM (0.7.5). Variants were identified using the HaplotypeCaller from GATK version 2.8.1 (Genome Analysis Toolkit, Broad Institute) along with Picard tools version 1.89. Sanger sequencing was performed for low coverage regions (GS-FLX: less than 15 reads and regions containing homopolymers > 5; SOLiD 5500 and MiSeq: less than 30 reads). Sample swap was ruled out by using a SNP check on an independent DNA dilution (genotyping at least 10 frequently occurring SNPs).

**Analytical performance of the next-generation platforms**

GS-FLX was validated by comparing Sanger and GS-FLX sequencing in DNA samples from 30 hypertrophic cardiomyopathy patients. The variants that were identified in the 7 overlapping genes were compared. In total 318 variants were detected with at least one platform (266 substitutions, 16 insertions of 1 or 2bp, and 36 deletions of 1, 2, or 5 bp). With Sanger sequencing two variants were missed, which yields a sensitivity of 99.3% (false positive rate of 0.7%). With GS-FLX a single variant was missed resulting in a sensitivity of 99.7% (false positive rate of 0.3%). For the validation of the SOLiD 5500 we tested whether 832 substitutions (304 different), 74 insertions and deletions previously detected with the GS-FLX, could be detected with the SOLiD 5500. The sensitivity of the SOLiD 5500 for all missense variants was 99.3% (826 of 832 variants were detected). The sensitivity for only the different missense variants was 99.0% (304 of 307 variants). Deletions <5 bp showed a sensitivity of 97.1% (33 of 34 variants). Deletions between 5 and 14 bp had a sensitivity of 100% (25 variants tested). Insertions <5 bp had a sensitivity of 100% (10 variants tested). Insertions > 5bp showed a sensitivity of 20% (only 1 of 5 was detected). The SOLiD system turned out to be not ideal for the detection of larger insertions therefore we switched to the MiSeq. For the validation of the MiSeq we compared 1242 substitutions (400 different), 34 different deletions and insertions between the Solid 5500 and the MiSeq. The sensitivity of the MiSeq for the missense variants was 100,2% (1242 of 1239 variants were detected). In addition during diagnostic testing we identified 252 class 3,4,5 variants with the MiSeq. All of them were confirmed by Sanger Sequencing. In total 1494 (1242+252) substitutions were correctly identified with the MiSeq (a false positive rate of 0%). With the Solid system we were able to correctly identify 2065 (1239+826) from the 2074 (832+1242) substitutions. A false positive rate of 0.4%. The sensitivity for only the different variants was 100,8% (400 of 397 variants). Deletions (between 1-53 bp) and insertions (between 1-68 bp) had a sensitivity of 94.1% (32 of 34 variants) on the MiSeq. We missed a 1 bp insertion and a 43 bp duplication that were present in low coverage regions. Since standardly the low coverage regions are additionally sequenced by Sanger sequencing these variants will not be missed in our combined (NGS+Sanger) test.

**Classification of variants**

Variant classification is performed by a molecular geneticist specialised in cardiogenetics and is checked by a second molecular geneticist. To classify the sequence variants we have used two scoring lists. Scoring list 1 was used for missense and other in-frame variants. Scoring list 2 was used for non-sense and frame-shift variants. For each variant we have analysed the outcome of many variant specific features. If a feature could not be calculated, we selected “not possible”. This score was not taken into account for the final classification. With these scoring lists the variants were classified in 5 different classes: Class 1: certainly not pathogenic, class 2: unlikely pathogenic, class 3: unknown pathogenicity, class 4: likely pathogenic; class 5: (certainly) pathogenic.

Scoring list 1 (see below) consists of two parts. In the first part variant specific features are calculated and scored based on in silico analysis using the variant interpretation software AlaMut (parts 1-6 and 9) and protein alignments as offered by AlaMut and/or homemade (part 6 and 7). All these features are basically based on conservation and the alteration in biophysical characteristics of the amino acid substitution. The score given to each feature is based on years of experience in interpreting sequence variants in autosomal dominant cardiac conditions in DNA diagnostics. Based on the presence and the frequency of the variant in a (preferably ethnically matched) control population a score is given (part 8 and table 1). For this we used Exome Aggregation Consortium (ExAC) database (http://exac.broadinstitute.org), GoNL (http://www.nlgenome.nl/) or other sources (in house, publications etc). In part 9 a score is given based on splice site prediction obtained by several splice site prediction software’s available in AlaMut. The scores obtained from the “in silico” part (1-9) are added and used to determine a sub-classification. Based on this information the highest score a variant can get is a Class 3 (favour class 4). Family information (co-segregation, literature, databases like HGMD professional), phenotypic features and/or functional analysis are needed to classify a variant as (likely) pathogenic (part 10 and 11).

Scoring list 2 (see below) consists also of two parts. The first part is based on splice predictions, general characteristics of the variant type and frequency in a control population (parts 1 till 3). Table 1 is used to determine the score with respect to frequency of the variant in controls if available (large deletions or duplications are not always called by annotation software). The scores obtained from the first part (1-3) are added and used to determine a sub-classification. Based on this information the highest score a variant can get is a Class 4. Family information (co-segregation), phenotypic features and/or functional analysis are needed to classify a variant as (likely) pathogenic (part 4 and 5). In addition if other variants (i.e. stop variant in about same region of the gene) with a comparable effect in that gene are known to be pathogenic for the disease. This variant can be classified as pathogenic.

**Scoring list 1 (missense and other in-frame variants)**

**1. a. HumDiv**

Probably damaging (score 1); Possibly damaging (score 0.5); Benign (score 0)

not possible or Score:……..

**b. HumVar**

Probably damaging (score 1); Possibly damaging (score 0.5); Benign (score 0)

**not possible or Score:……..**

**2. SIFT**

Score 1: Deleterious(0.00-0.05); Score 0: Tolerated (>0.05)

**not possible or Score: …..**

**3. Grantham dist (0-215)**

Score 2: Large distance (>140); Score 1: Moderate distance (≤140); Score 0: Benign (≤70)

**not possible or Score: …..**

**4. Align-GVGD**

Score 1.25: Class C65; Score 1: Class C55; Score 0.75: Class C45; Score 0.5: Class C35; Score 0.25: Class C15/25; Score 0: Class C0

**not possible or Score: …..**

**5. Blosum 62**

Score 1: ≥ -2; Score 0.5: -1; Score 0: ≥0

**not possible or Score: …..**

**6. Conservation between species using protein alignments** (use at least human, 3 other mammals and 3 lower animals like bird, frog, fly, fish)

Score 1 (All mammals and almost all lower animals); Score 0,75 (All mammals and a few lower animal); Score 0.5 (Almost all mammals and no lower); Score 0 (other)

**not possible or Score: …..**

**7. Conservation between isoforms (different genes) using protein alignments**

Score 0.5 (75-100%); Score 0.5 (35-74%); Score 0.25 (0-34%); Score 0 (0%)

**not possible or Score: …..**

**8. Frequency in control population** (Determine the score based on table 1)

**a.** Ethnical background of the patient matches the control population:

Yes; Unknown; No

**b.** Exome Aggregation Consortium (ExAC) database (http://exac.broadinstitute.org):

1. …….variant alleles in ……total alleles (all controles): allel freq: .…….%

2. …….variant alleles in ……total alleles (Ethnically matched controles): allel freq .…….%

**c.** Other sources (GoNL, in house databases, literature, ….): ..……………………….

**not possible or Score: …..**

**9. In silico analysis of splicing** (Splice prediction using AlaMut)

Score 2: probably functional (group 4); Score 1: possibly functional (group 3); Score 0.5: not likely (group 2); Score 0: unlikely (group 1)

**not possible or Score: …..**

**Total score for 1 till 9: …….**

**Maximum score possible*: …….**  (“not possible” is excluded)

**10. Family information/Phenotype?** (the information can also come from other families or literature)

Summarize all available information and evidence (e.g. literature references).

Score 4 (Very likely pathogenic): de novo variant or ≥6 affected family members with the variant and no affected

Score 3 (probably co-segregation): 5 affected family members with the variant and no affected without the variant*

Score 2 (possible co-segregation): 3-4 affected family members with the variant and no affected without the variant*

Score 1: (co-segregation unclear): 2 affected family members with the variant and no affected without the variant*

Score 0: only index/proband or no co-segregation (affected family member without variant)

**Score: …..**

*This does not count when the phenotype is likely due to a non genetic cause like hypertension in cardiac hypertrophy or when it is likely that more than a single variant explains the phenotype in a severely affected patient.

**11. Functional analysis**

Experimentally:

Is the variant functionally tested in vitro, in culture or in an animal model? If so judge based on the method used and the experimental data how convincing the conclusion is. This is important because functional assays are often not well validated.

Summarize all available information and evidence (e.g. literature references).

Score 3: Convincingly functionally aberrant

Score 1: Possibly functionally aberrant

Score 0: Unclear or not functionally aberrant

**not possible or Score: …..**

**Sub-classification based on parts 1-9: Sub-class …**

Calculate % score= 100* (total score for 1 till 9/ maximum score possible for 1 till 9) = …. %

% score ≥ 70%: Class 3 (favor class 4; at least 5 parts should give informative information)

45% ≤ % score < 70%: Class 3

25% ≤ % score < 45%: Class 2

% score < 25%: Class 1 not pathogenic*

*missense variants which get a sub-classification “not pathogenic” but were observed between 0.02 and 0.3% in a large population of control alleles will be upgraded to a Class 2. Missense variants that are rare <0.02% and are only found in <3 index patients (no co-segregation information available, or functional data) are classified as a class 3 (irrespective of the sub-classification).

**Final classification (including part 10 and 11):**

Family information (co-segregation), phenotypic features and/or functional analysis are needed to classify a variant as (putative) pathogenic.

1. A combined score of 2 or 3 for part 10 and 11 will upgrade the score from the sub-classification one level.

2. A maximum score for part 11 (functional analysis) and a score 0 for part 10 (Family information) upgrades every sub-class to a Class 4 (a functional test on its own is not enough to give a variant the classification pathogenic).

3. A combined score of 4 for part 10 and 11 and none of the parts have a maximum score will upgrade the score from the sub-classification to a Class 4.

4. When in part 10 a maximum score is obtained the sub-classification is upgraded to a pathogenic variant.

5. A combined score of 5 or 6 for part 10 and 11 and part 10 has not the highest score will upgrade the score from the sub-classification to a pathogenic variant.

**Score part 10 (family information): …….**

**Score part 11 (functional analysis): …….**

**Total score parts 10 & 11: ..…**

**Conclusion: Class …**

Class 1: certainly not pathogenic, class 2: unlikely pathogenic, class 3: unknown pathogenicity, class 4: likely pathogenic; class 5: (certainly) pathogenic.

Comment:

If the scoring list is not in agreement with other information not included in this list indicate this below and correct the conclusion accordingly.

**Scoring list 2 (non-sense and frame-shift variants)**

**1. Non-sense variant or predicted influence on splicing (table 2)**

Score 4: c.x-1 or -2 (acceptor) or c.y+1 or +2 (donor) and clear reduction (loss of) of splice site predicted values in AlaMut or stop or frameshift variant*

New or altered splice site predicted (see table 2)

Score 2: probably functional (group 4); Score 1: possibly functional (group 3); Score 0.5: not likely (group 2); Score 0: unlikely (group 1)

**not possible or Score: …..**

* When the stop or frameshift variant is in the last 2 exons this may result in a stable protein and if the N-terminal part of the protein is not well conserved it remains uncertain whether the variant will be pathogenic.

**2. Does the variant type fit with the disease?** (Think about gain or loss of function, dominant negative, haplo-insufficiency etc.)

Score 5: Type of variant fits with the disease

Score 1: Type of variant not described before in disease

Score 0.5: Unlikely disease causing

(Not) pathogenic because:….……………………………………………………………

**not possible or Score: …..**

**3. Frequency in control population** (Determine the score based on table 1)

**a.** Ethnical background of the patient matches the control population:

Yes; Unknown; No

**b.** Exome Aggregation Consortium (ExAC) database (http://exac.broadinstitute.org):

1. …….variant alleles in ……total alleles (all controles): allel freq: .…….%

2. …….variant alleles in ……total alleles (Ethnically matched controles): allel freq .…….%

**c.** Other sources (GoNL, in house databases, literature, ….): ..……………………….

**not possible or Score: …..**

**4. Family information/Phenotype?** (the information can also come from other families or literature)

Summarize all available information and evidence (e.g. literature references).

Score 4 (Very likely pathogenic): de novo variant or ≥6 affected family members with the variant and no affected

Score 3 (probably co-segregation): 5 affected family members with the variant and no affected without the variant*

Score 2 (possible co-segregation): 3-4 affected family members with the variant and no affected without the variant*

Score 1: (co-segregation unclear): 2 affected family members with the variant and no affected without the variant*

Score 0: only index/proband or no co-segregation (affected family member without variant)

**Score: …..**

*This does not count when the phenotype is likely due to a non genetic cause like hypertension in cardiac hypertrophy or when it is likely that more than a single variant explains the phenotype in a severely affected patient).

**5. Functional analysis**

Experimentally:

Is the variant functionally tested in vitro, in culture or in an animal model? If so judge based on the method used and the experimental data how convincing the conclusion is. This is important because functional assays are often not well validated.

Summarize all available information and evidence (e.g. literature references):

Score 3: Convincingly functionally aberrant

Score 1: Possibly functionally aberrant

Score 0: Unclear or not functionally aberrant

**not possible or Score: …..**

**Sub-classification based on parts 1-3: Sub-class …**

Calculate % score: 100* (total score for 1 till 3/ Maximum score possible for 1 till 3) = …. %

% score ≥ 70%: Class 3 (favour class 4; at least 5 parts should give informative information)

45% ≤ % score < 70%: Class 3

25% ≤ % score < 45%: Class 2

% score < 25%: Class 1 not pathogenic*

Silent variants without (or with hardly) an effect on splicing in AlaMut are classified as class 1 (irrespective of occurrence in control population).

**Final classification (including part 4 and 5):**

Family information (co-segregation), phenotypic features and/or functional analysis are needed to classify a variant as (putative) pathogenic.

1. A combined score of 2 or 3 for part 4 and 5 will upgrade the score from the sub-classification one level.

2. A maximum score for part 5 and a score 0 for part 4 upgrades every sub-class to a Class 4 (a functional test on its own is not enough to give a variant the classification pathogenic).

3. A combined score of 4 for part 4 and 5, and none of the parts have a maximum score, will upgrade the score from the sub-classification to a Class 4.

4. When in part 4 a maximum score is obtained the sub-classification is upgraded to a pathogenic variant.

5. A combined score of 5 or 6 for parts 4 and 5, and part 4 has not the highest score, will upgrade the score from the sub-classification to a pathogenic variant.

**Score part 4 (family information): …….**

**Score part 5 (functional analysis): …….**

**Total score parts 4 & 5: ..…..**

**Conclusion: Class ……**

Class 1: certainly not pathogenic, class 2: unlikely pathogenic, class 3: unknown pathogenicity, class 4: likely pathogenic; class 5: (certainly) pathogenic.

Comment:

If the scoring list is not in agreement with other information not included in this list indicate this below and correct the conclusion accordingly.

Table 1: Score table for the frequency of the variant in control alleles (>1500 alleles)

| **Variant allele frequency (%)** | **Match in ethnical background** | **Score** |
| --- | --- | --- |
| Not present | Yes | 2 |
|  | Unknown | 2 |
|  | No | 1 |
| 0<freq≤0.02 | Not important | 1.5 |
| 0.02<freq≤0.05 | Not important | 1 |
| 0.05<freq≤0.1 | Not important | 0.5 |
| >0.1 | Not important | 0 |
| > 0.3* | Not important | Not pathogenic |

*for a autosomal dominant disease (the variant should not be known as a founder mutation).

**Table 2: Classification putative splice-site variants using splice site predictions** in AlaMut

|  | | | Number of predicted sites |
| --- | --- | --- | --- |
| wt (genuine) splice site | | | …. |
| Putative splice site | | | …. |
|  | | | |
| Value putative site compared to wt (in %): | | | |
| SpliceSiteFinder-like | …. % of wt | | |
| MaxEntScan | …. % of wt | | |
| NNSPLICE | …. % of wt | | |
| GeneSplicer | …. % of wt | | |
| Human Splice Finder | …. % of wt | | |
| **Prediction of a splice site that is normally not used** | | | |
| Group 1 (unlikely) | | 1 program predicts putative site or | |
|  | | highest probability putative sites <70% of wt | |
| Group 2 (not likely) | | ≥ 2 programs predict putative site and | |
|  | | highest value for one program 70%≤wt<90% | |
| Group 3 (possibly functional) | | ≥ 2 programs predict putative site and | |
|  | | highest value for one program ≥90% of wt or | |
|  | | highest values for ≥2 programs 70%≤wt<90% | |
| Group 4 (probably functional) | | ≥ 2 programs predict putative site and | |
|  | | highest value for ≥ 2 programs ≥90% of wt | |
| **Change of predicted values of the genuine splice site** | | | |
| Group 1 (unlikely) | | 3 programs >90% of wt and other programs 50<wt< 90% | |
| Group 2 (not likely) | | 2 programs >90% of wt and other programs 50<wt< 90% | |
| Group 3 (possibly functional) | | 1 program >90% of wt and other programs ≤50% of wt | |
| Group 4 (probably functional) | | 1 program <90% and other programs ≤50% of wt | |
| Group 5 (functional) | | c.x-1 or -2 (acceptor) or c.y+1 or +2 (donor)  and clear reduction (loss) of genuine splice site predicted values in AlaMut | |

% arbitrarily chosen
