# Supplementary material for: Large next-generation sequencing gene panels in genetic heart disease: yield of pathogenic variants and variants of unknown significance
Source: Neth Heart J. 2019 Mar 7;27(6):304–9. doi: 10.1007/s12471-019-1250-5 (PMC6533346; doi:10.1007/s12471-019-1250-5)
Supplement: Supplementary file 3 — Detected known Dutch founder variants and other recurrent possible founder variants (all class 5) [file 12471_2019_1250_MOESM3_ESM.docx]

| **Online Supplementary File 3** Detected known Dutch founder variants and other recurrent possible founder variants (all class 5) | | |
| --- | --- | --- |
| **Gene** | **Variant** | **Frequency (*n*)** |
| *Known founder variants* |  |  |
| DPP6^1^ | Risk haplotype | 5 |
| KCNQ1^2^ | c.887T>C; p.(Phe296Ser) | 2 |
| MYBPC3^3^ | c.2373dup; p.(Trp792Valfs*17) | 46 |
| MYBPC3^3^ | c.2827C>T; p.(Arg943*) | 13 |
| MYBPC3^3^ | c.2864_2865del; p.(Pro955Argfs*95) | 4 |
| PKP2^4^ | c.235C>T; p.(Arg79*) | 2 |
| PLN^5^ | c.40_42del; p.(Arg14del) | 21 |
| TNNI3^6^ | c.433C>T; p.(Arg145Trp) | 1 |
| TNNI3^6^ | c.497C>T; p.(Ser166Phe) | 2 |
| TNNI3^6^ | c.626A>C; p.(Glu209Ala) | 2 |
| TNNT2^7^ | c.650_652del; p.(Lys217del) | 2 |
| *Recurrent variants* |  |  |
| DSP | c.1266+1G>T | 3 |
| HCN4 | c.1471G>T; p.(Asp491Asn) | 3 |
| LMNA | c.992G>A; p.(Arg331Gln) | 7* |
| MYBPC3 | c.442G>A; p.(Gly148Arg) | 5* |
| MYBPC3 | c.481C>T; p.(Pro161Ser) | 3 |
| MYBPC3 | c.897del; p.(Lys301Argfs*49) | 4 |
| MYBPC3 | c.927-2A>G | 4 |
| MYBPC3 | c.1484G>A; p.(Arg495Gln) | 5 |
| MYBPC3 | c.1831G>A; p.(Glu611Lys) | 3 |
| MYBPC3 | c.2149-2del | 2 |
| MYBPC3 | c.2308+1G>A | 2 |
| MYBPC3 | c.3190+5G>A | 2 |
| MYBPC3 | c.3628-41_3628-17del | 4 |
| MYBPC3 | c.3776del; p.(Gln1259Argfs*72) | 3 |
| MYH7 | c.1208G>A; p.(Arg403Gln) | 3 |
| MYH7 | c.1987C>T; p.(Arg663Lys | 3 |
| MYH7 | c.2609G>A; p.(Arg870His) | 2 |
| MYH7 | c.3100-2A>C | 2 |
| MYH7 | c.5754C>G; p.(Asn1918Lys) | 3 |
| MYL2 | c.64G>A; p.(Glu22Lys) | 4 |
| PKP2 | c.397C>T; p.(Gln133*) | 4 |
| PRKAG2 | c.905G>A; p.(Arg302Gln) | 2 |
| RBM20 | c.1900C>T; p.(Arg634Trp) | 4 |
| SCN5A | c.795_808del; p.(Leu266Alafs*40) | 2 |
| SCN5A | c.2582_2583del; p.(Phe861Trpfs*90) | 4 |
| SCN5A | c.3956G>T; p.(Gly1319Val) | 3 |
| SCN5A | c.4975A>G; p.(Asn1659Asp) | 2 |
| SCN5A | c.5228G>A; p.(Gly1743Glu) | 2 |
| TNNI3 | c.626A>C; p.(Glu209Ala) | 2 |
| TNNT2 | c.277G>A; p.(Glu93Lys) | 2 |
| TNNT2 | c.304C>T; p.(Arg102Trp) | 4 |
| TNNT2 | c.650_652del; p.(Lys217del) | 2 |
| TTN | c.52222+1G>A | 3 |
| TTN | c.78936C>A; p.(Tyr26312*) | 3 |
| * Class 5 variant after reclassification.  ^1^ Postema PG, Christiaans I, Hofman N, et al. Founder mutations in the Netherlands: familial idiopathic ventricular fibrillation and DPP6. Neth Heart J. 2011;19:290-6.  ^2^ Hofman N, Jongbloed R, Postema PG, et al. Founder mutations in the Netherlands: the Long-QT Syndrome. Neth Heart J.2011;19:10-16.  ^3^ Christiaans I, Nannenberg EA, Dooijes D, et al. Founder mutations in hypertrophic cardiomyopathy in the Netherlands. Neth Heart J. 2010;18:248-54.  ^4^ Van der Zwaag PA, Cox MGPJ, van der Werf C, et al. Recurrent and founder mutations in the Netherlands: Plakophilin-2 p.Arg79X mutation causing arrhythmogenic right ventricular cardiomyopathy/dysplasia. Neth Heart J. 2010;18:583-91.  ^5^ Van der Zwaag PA, van Rijsingen IAW, de Ruiter R, et al. Recurrent and founder mutations in the Netherlands – Phospholamban p.Arg14del mutation causes arrhythmogenic cardiomyopathy. Neth Heart J. 2013;21:286-93.  ^6^ Van den Wijngaard A, Volders P, van Tintelen JP, et al. Recurrent and founder mutations in the Netherlands: cardia Troponin I (TNNI3) gene mutations as a cause of severe forms of hypertrophic and restrictive cardiomyopathy. Neth Heart J. 2011;19:344-51.  ^7^ Otten E, Lekanne dit Deprez RH, Weiss MM, et al. Recurrent and founder mutations in the Netherlands: mutation p.K217del in troponin T2, causing dilated cardiomyopathy. Neth Heart J. 2010;18:478-85.  Almost all recurrent mutations have been described before in literature. | | |
